# Urban Nature Experiences Reduce Stress in the Context of Daily Life Based on Salivary Biomarkers

**DOI:** 10.3389/fpsyg.2019.00722

**Published:** 2019-04-04

**Authors:** MaryCarol R. Hunter, Brenda W. Gillespie, Sophie Yu-Pu Chen

**Affiliations:** ^1^School for Environment and Sustainability, University of Michigan, Ann Arbor, MI, United States; ^2^Consulting for Statistics, Computing, and Analytics Research, University of Michigan, Ann Arbor, MI, United States; ^3^Department of Biostatistics, University of Michigan, Ann Arbor, MI, United States

**Keywords:** nature pill, stress reduction, adaptive intervention, cortisol, amylase, mental well-being, duration prescription, affordable healthcare

## Abstract

Stress reduction through contact with nature is well established, but far less is known about the contribution of contact parameters – duration, frequency, and nature quality. This study describes the relationship between duration of a nature experience (NE), and changes in two physiological biomarkers of stress – salivary cortisol and alpha-amylase. It is the first study to employ long-term, repeated-measure assessment and the first evaluation wherein study participants are free to choose the time of day, duration, and the place of a NE in response to personal preference and changing daily schedules. During an 8-week study period, 36 urban dwellers were asked to have a NE, defined as spending time in an outdoor place that brings a sense of contact with nature, at least three times a week for a duration of 10 min or more. Their goal was compliance within the context of unpredictable opportunity for taking a nature pill. Participants provided saliva samples before and after a NE at four points over the study period. Before-NE samples established the diurnal trajectory of each stress indicator and these were in line with published outcomes of more closely controlled experiments. For salivary cortisol, an NE produced a 21.3%/hour drop beyond that of the hormone’s 11.7% diurnal drop. The efficiency of a nature pill per time expended was greatest between 20 and 30 min, after which benefits continued to accrue, but at a reduced rate. For salivary alpha-amylase, there was a 28.1%/h drop after adjusting for its diurnal rise of 3.5%/h, but only for participants that were least active sitting or sitting with some walking. Activity type did not influence cortisol response. The methods for this adaptive management study of nature-based restoration break new ground in addressing some complexities of measuring an effective nature dose in the context of normal daily life, while bypassing the limitations of a clinical pharmacology dose–response study. The results provide a validated starting point for healthcare practitioners prescribing a nature pill to those in their care. This line of inquiry is timely in light of expanding urbanization and rising healthcare costs.

## Introduction

Exposure to nature has great benefits (Hartig et al., 2011; Ward Thompson, 2011; Bratman et al., 2012; Haluza et al., 2014; van den Bosch and Ode Sang, 2017), key among them being a better state of mental well-being (for example, Berman et al., 2008; Logan and Selhub, 2012; Hartig et al., 2014; Bratman et al., 2015; Hansen et al., 2017). While many studies show a positive influence of nature exposure on health and well-being, there is little understanding about how much or in what form a nature experience (NE) should be for best effect. Healthcare providers in North America and Europe have begun to write nature prescriptions, often called “nature pills,” using common sense and interpretation of published research to motivate patients to take a nature break (James et al., 2017; Wessel, 2017). Likewise civic organizations and non-profits are emerging in support of the nature-well-being treatment such as the Mood Walks program in Canada^1^, the Nature Sacred program of the TKF Foundation^2^ in the United States, the Dose of Nature project in the United Kingdom (Bloomfield, 2017), and the Coastrek program in Australia (Buckley et al., 2016). Laudable examples aside, there are no quantitative studies on the frequency of nature pill prescribing and what exactly is being dispensed. There is a clear need for research that specifies the parameters of a nature pill that best support mental health. This line of inquiry on pro-active healthcare is timely in light of rising healthcare costs worldwide and the impact of growing urbanization that limits access to nature (WHO, 2016).

Dose–response approaches to the study of the nature-well-being relationship have recently been used to quantify how much and what kind of nature produces positive effects on human well-being. Theoretical frameworks for hypothesis testing and operationalizing methods have been put forward to identify the impact of duration, frequency, and intensity of a NE on health and well-being (Sullivan et al., 2014; Hunter and Askarinejad, 2015; Shanahan et al., 2015; Frumkin et al., 2017; Van den Berg, 2017). Shanahan et al. (2015) charted a framework for a more holistic consideration of what to account for in dose–response studies. The authors also discuss the key attributes of a dose–response model to identify threshold dose recommendations for specific health outcomes. Like others, they conclude that most research on parameters of a nature pill are too coarse, for example, urban versus “natural” settings, percent of foliage or green space in sight or nearby, duration of the nature interface is set by the researcher, and experiments that are often done indoors in lab settings. Frumkin et al. (2017) offer a research agenda on nature contact-health relationships while detailing the complexities of quantifying “dose.”

Empirical approaches to the study of the nature dose–well-being response relationship are varied and have provided rich ways to deconstruct how the duration, frequency, and intensity of a nature dose contribute to physical and mental well-being, and how social, economic, and demographic factors adjust a dose–response relationship (Jiang et al., 2014; Shanahan et al., 2016; Cox et al., 2017a; Frumkin et al., 2017). In population-level studies, spatially grouped measures of human health and well-being are interpreted relative to the amount (dose) of nearby nature, for example, street tree density per 17 ha (Kardan et al., 2015), tree canopy per postal code (Cox et al., 2017b), and degree of urbanization per postal code (Cox et al., 2018).

Our ultimate goal is to articulate a “nature prescription” for use by healthcare providers as a preventive, self-administered health care treatment for mental well-being that is low in cost and effective in everyday settings. Full articulation of a prescription involves knowing the efficacy of which pill, at what dose, and how often. From this broad arena, we chose to start by examining the duration aspect of efficacy using objective assessment of physiological stress.

The dose–response for duration of nature exposure has been measured in a variety of ways, with the subjective assessment of mental state dominating (e.g., mood, ability to focus, and perceived level of stress, anxiety, or contentment). The subjective nature of self-report data for professional healthcare treatment decisions is considered less desirable than objectively sourced data (Van den Berg, 2017), such as change in blood pressure, heart rate, and stress hormone level. Consequently, we chose two biomarkers of physiological stress – salivary cortisol and salivary alpha-amylase, to quantify the change in physiological stress in response to the duration of nature exposure. In nature restoration studies, the hormone cortisol has proven to be an attractive biomarker of stress as it is sampled in a relatively non-invasive way through saliva collection (e.g., Ward Thompson et al., 2012; Jiang et al., 2014; Gidlow et al., 2016).

The utility of cortisol and amylase as biomarkers is predicated on being able to separate the nature exposure effect from the natural diurnal shift in production. Moreover, cortisol and amylase have distinct diurnal patterns. Salivary cortisol is highest in the morning after a brief pulse upon awakening and then drops through the day and into the night. By contrast, amylase has a distinct drop in the first hour after waking and a steady increase toward evening (Nater et al., 2007). The circadian rhythm of cortisol production in humans has seasonal shifts being lower in summertime, coinciding with earlier sunrise time (Hadlow et al., 2014). For amylase, the impact of day length is unstudied in humans, but in rats, the diurnal rhythm of alpha-amylase production changes under different photoperiod treatments (Bellavia et al., 1990). Collectively, these findings underscore the need to account for time of day for both biomarkers when interpreting the body’s response to nature interventions. Most researchers accommodate the diurnal shifts by conducting experiments at roughly the same time of day, assuming the bias of diurnal change will be of equal impact regardless of day length and across participants regardless of treatment group. This approach has merit but limits what can be learned and might lead to mistaken conclusions.

Published studies using physiological criteria to investigate the impact of NE on stress are typically based on a single fixed duration time, with 15 and 30 min being the most common over a range of 10–90 min. Conclusions about the ability of a NE to influence well-being emerge from relative comparisons (paired *t*-test or analysis of variance) between the treatment (NE) and a control (typically an intensely urban experience). Consequently, none of these studies can be used to interpret threshold effects – minimum time for a nature pill effect, or duration-based efficacy. What is needed is a sampling method that provides nature response data over a continuum of duration times.

In terms of experimental design, randomized clinical trials (RCTs) are the gold standard for objective information on optimal dosing/exposure and the effectiveness of different types of intervention for different user groups. But, investigations about the restorative value of nature exposure are generally unsuited for the exacting protocol of an RCT. The most unavoidable conflict arises when participants and researchers cannot be blinded to the identity of the intervention, thereby introducing a perception bias (Van den Berg, 2017). Another challenge is achieving participant compliance with a behavior-based intervention (Olem et al., 2009) that (a) must be managed within the messy context of daily life, (b) is in the realm of preventive care (versus acute care), and (c) requires more time and effort than just taking a pill.

In developing an alternative approach to the RCT, our experimental design was inspired by the research of Collins et al. (2004) and Murphy et al. (2007) on adaptive intervention strategies to prevent and treat conditions that have behavioral components such as mental illness and substance abuse. Here, the course of treatment is adjusted in real time in response to what does and does not work for the individual. The behavior adaptability aspect of this approach is well suited to our goal of measuring the impact of self-directed nature exposure on mental well-being in the context of daily life for a healthy-normal population. The key difference is that our participants develop their own set of decision rules to comply with a prescribed minimum of three nature pills per week.

The experimental design presented here allows the participant to adjust terms of the nature intervention (duration, nature quality, and when it happened) for their convenience, while abiding by a set of ground rules. The goal of this adaptive intervention strategy is to introduce variation in nature pill duration and to embrace the backdrop of stress variation in daily life for a realistic estimate of effective dose. During the 8-week experiment, participants were asked to maintain a behavior regime of 3 NEs a week. Over the 2-month period, there were four tests of physiological stress, taken at the discretion of the participant (although they were asked to do so approximately every 2 weeks). Throughout the experimental period, each participant was able to customize the nature intervention in response to the constraints and unpredictability of real life by having control of the date, time of day (anytime from 1 h after rising until nightfall), and duration (10 min or more) of the NE. Use of this adaptive approach also intends to reduce some of the motivation problems that are inherent to interventions that demand greater effort and more planning than taking a pill on schedule.

The protocols for personally customized nature pills produced data that required a different analytical approach. In other stress studies using cortisol and amylase markers, the diurnal change in cortisol (daytime falling) and amylase (daytime rising) is accommodated in one of two ways. Either the participant is sampled repeatedly through the preceding day or the day of the intervention (and typically in clinical settings, for example, Rohleder and Nater, 2009) to establish the personal diurnal slope, or it is assumed that stress testing at the same time of day eliminates the contribution of diurnal change (e.g., Hansen et al., 2017). Neither protocol was useful for an experimental design based on adaptive management for self-care. To estimate a reliable nature pill prescription for members of the normal, healthy population, we present a new approach to accommodate diurnal fluctuation in the stress markers.

Our experimental goal is to investigate how relatively a short NE influences stress level within the context of everyday life using objective physiological indicators of stress. We use a repeated measures experimental design to (1) estimate the duration of an effective nature pill; (2) evaluate the impact of a NE on stress under conditions typical of the participant’s daily life using an adaptive management approach; and (3) distinguish the diurnal response of the stress markers from the nature pill effect. Because proactive health care is foundational to reducing health care costs, another goal is to identify experimental approaches that improve the time and cost efficiency of research about behavioral self-care in support of better mental health.

## Materials and Methods

### Selection of the Stress Biomarkers

The definition of stress varies among fields and specialties depending on what’s in focus – perception of stress, behavioral response to stress, and neurophysiological response to stress. This study investigated the latter, using two known physiological biomarkers of stress. Like a pharmaceutical under test, the treatment – a NE, was evaluated for its competency as de-stressor rather than a stressor. The established biomarkers of stress – salivary cortisol and salivary alpha-amylase (hereafter, cortisol and amylase), have different pathways. The autonomic nervous system initiates adjustment to stress with signals from the hypothalamic pituitary adrenal (HPA) axis which controls cortisol, and the sympathetic adrenal medullary (SAM) axis which controls amylase (Kirschbaum and Hellhammer, 1994; Nater and Rohleder, 2009). These biomarkers are easily sampled in field settings using non-invasive, self-administered collection of saliva samples (Yamaguchi and Shetty, 2011; Nater et al., 2013b).

Cortisol is the primary stress hormone. It mediates the physical pathways of many metabolic processes involved with homeostasis including that of immune function. Prolonged elevation of cortisol interferes with learning and memory, lowers immune function and bone density, and increases blood pressure, cholesterol, heart disease, and weight (McEwen, 2008; Lupien et al., 2009). In a study of demographic and socioeconomic differences in daytime trajectories of cortisol, Karlamangla et al. (2013) analyzed data from the Midlife in the United States Study (MIDUS), and found differences in the diurnal rhythm of salivary cortisol based on age, gender, ethnicity, and education. They conclude that sampling cortisol of each participant over multiple days of a study (four times in this case) would ensure a better capture of the daytime diurnal cycle to explain some of the variation owing to differences in waking time, sleep duration, and workday versus weekend day status.

Salivary amylase is an enzyme produced by the digestive system. It is responsive to both physical and psychological stressors (Nater et al., 2007; Breines et al., 2015) and is used increasingly for stress evaluation portrayed by the sympathetic nervous system – SAM (Nater and Rohleder, 2009). Amylase is a useful marker to investigate the stress response to physical stressors (e.g., exercise, Koibuchi and Suzuki, 2014) and mental stressors (e.g., psychosocial distress (Rohleder et al., 2004; Obayashi, 2013). Amylase is also used to study of value of interventions for stress relief, most often involving physically passive interventions such as listening to music or reading (e.g., Linnemann et al., 2015). There are four studies on the impact of outdoor experience on amylase response (Kondo et al., 2018).

For amylase, there are no gender differences in diurnal response (Nater et al., 2007), although research on the complexity of the gender factor regarding the biological stress response has yet to provide a clear resolution on this point for either cortisol or amylase (Strahler et al., 2017). Research about age-based changes in amylase production has mixed outcomes regarding diurnal changes and response to stress (Rohleder and Nater, 2009), but it is clear that the stress response is different for very young children and the elderly (Strahler et al., 2017). Amylase production is much more sensitive to environmental input than is cortisol. For example, amylase is stimulated by caffeine, food or chewing itself, and exercise, and is inhibited by smoking, chronic drinking, some medicines (Nater et al., 2007). Consequently, it is important to control or account for interference from those environmental variables known to adjust amylase response.

### Study Group

Participants were recruited via email announcements and flyers directed to faculty and staff at the University of Michigan and members of several local non-profit organizations in Ann Arbor, MI, United States. Recruitment focused on members of the normal, healthy population, age 18 and over, interested in spending more time outdoors in green spaces. Participants were self-selected. A minimum sample size of 30 was calculated (SAS^®^ software, version 9.4, SAS Institute Inc., Cary, NC, United States) based on having 86% power to detect an *R*^2^ of 0.25 using linear regression. The study had approval from the Institutional Review Board of the University of Michigan, Ann Arbor, MI, United States (IRB # HUM00089147).

In all, 44 participants were recruited, and 36 provided sufficient reliable data. Of these 92% were female (33). The mean age overall was 45.8 years (SD = 13.35, range 22–68). The sample was 86% white (31) of which two were of Hispanic ethnicity, 6% Asian (2), and 8% all others (3). Thirty-six participants had an average of 3.22 NEs (out of four, SD = 0.87).

### Sampling Scheme

In accordance with our goal of defining a nature prescription for everyday life, the experimental design let participants use adaptive management to better support the behavior of taking a nature pill. Participants completed an 8-week summer study starting in mid-June 2014. The goal was to have a NE at least three times a week on days of their choice. During a NE, they could sit, walk, or do both in an outdoor location of their choice. The NE was defined as *anywhere outside that, in the opinion of the participant, included a sufficiency of natural elements to feel like a nature interaction*. Participants understood they were free to adjust the place, time of day, and duration of the NE in response to changing daily circumstances to best accommodate their goal.

The ground rules stipulated the following. The saliva sampling must take place in daylight, at least 1 h after waking and be completed before nightfall. For the 30 min before saliva was taken, there could be no eating, drinking, or toothpaste. The NE itself could not include aerobic exercise in order to limit the opportunity for an exercise-based rise in endocannabinoids. Use of social media, the internet, phone calls, conversations, or reading were also to be avoided.

### Collection and Analysis of Saliva Samples

Participants provided saliva samples just before and just after a NE on 4 days during the 8-week experimental period. They were encouraged to do this at the end of the first, third, fifth, and seventh week. The collection period ran from June 17 to August 21, 2014 with a median sampling date of July 22, 2014. Almost all NEs took place in the Ann Arbor, Michigan area. Over this period, sunrise in Southeast Michigan occurred between 5:58 and 6:49 a.m. and sunset occurred between 8:47 and 9:14 p.m.^3^. Prior to each NE, (pre-NE) samples were collected as early as 7:03 a.m. and as late as 11:28 p.m.; some samples were taken outside the requested window of “before nightfall.”

Training for saliva collection took place at orientation meetings where participants signed consent forms at the outset. Each participant received a small carrier with a pouch that held a blue ice pack and four pairs of Salivette tubes (produced by Sarstedt Inc.) with labels titled BEFORE or AFTER. During orientation, participants under supervision did a test run (without the NE, but with two saliva collections at least 10 min apart) using the following protocol. Just before a NE, the participant placed a cotton roll from the BEFORE Salivette tube into their mouth, chewed on it for 1–2 min (until fully wet), returned the cotton roll to the BEFORE tube, re-capped, and labeled it with their unique three-letter ID plus date and time. The process was repeated just after the NE ended using the vial labeled AFTER. At this point, the participant reported whether they had been “sitting,” “sitting and walking”, “walking,” or “other” during the NE. When “other” was chosen, the activity type was specified. Participants also answered questions about their compliance (or lack of it) with the ground rules listed above.

Although saliva can be stored at room temperature for up to 3 weeks, freezing or at least refrigeration will prevent mold and bacterial growth (Rohleder and Nater, 2009). Consequently, participants were asked to store samples in a home or office freezer until the 8-week testing period was over at which point their entire sample set was delivered to the lab in the carrier with an ice pack. Thereafter, samples were stored at -20°C. All protocols regarding thermal conditions of samples during transport and storage are in keeping with good practice for stability of cortisol (Garde and Hansen, 2005; Nalla et al., 2015) and amylase (Rohleder and Nater, 2009).

Frozen samples were assayed in a single batch 1 month after the close of the study for cortisol and 8 months after the study close for amylase. Concentrations of salivary cortisol and salivary alpha-amylase were measured at the Core Assay Facility, University of Michigan’s Department of Psychology using commercially available kits from Salimetrics. Each assay was run in duplicate. Cortisol level was reported as μg/dL. Amylase level was reported in enzyme units per milliliter (U/mL), a number that reflects the amount of enzyme that catalyzes the conversion of 1 mmol of substrate per minute (Rohleder and Nater, 2009). The inter-assay coefficient of variation (CV) was 34% for cortisol and 65% for amylase. The intra-assay CV was 7.0% for cortisol and 3.7% for amylase. Analytical sensitivity for both stress markers was <0.007 μg/dL according to the kit manufacturer. All duplicate measures passed the check for similarity.

### Statistical Analyses

The presence of diurnal cycles in cortisol and amylase highlighted the need to account for time of day. Consequently, a novel approach for accommodating time of day was developed and adopted. The samples taken pre-NE collectively established the diurnal trajectory of the study population. A comparison of stress indicator levels before (diurnal component) and after a NE (diurnal + NE components) allowed interpretation about the impact of the NE with the natural cycles of diurnal shift in biomarkers accounted for. We also investigated effects of participant activity types (sitting and walking), as well as the effect of nightfall onset on cortisol and amylase levels. Finally, the efficiency of a NE in reducing stress relative to NE duration was evaluated. A mixed model regression approach accounted for multiple measurements per participant. The model was used to establish the diurnal trajectory and to evaluate the effect of a NE on stress. Biomarkers were log-transformed to adjust for the non-linearity in each diurnal cycle. All analyses were conducted using SAS^®^ software, version 9.4 (SAS Institute Inc., Cary, NC, United States) unless otherwise specified.

Sample exclusions were as follows. For seven NEs, at least one of the two paired outcomes (the pre-NE and post-NE samples) was not eligible. The amylase assay result was missing for two NEs, one a pre-NE and one a post-NE, so the existing data from the NE pair member was removed from consideration. Two amylase samples with levels greater than three SDs from the mean (>500 U/mL) were removed as were their NE pair members. One participant reported bicycling as the activity during three NEs. The pre and post samples for each were removed because of the capacity of aerobic exercise to influence amylase production.

## Results

Both stress biomarkers indicated a reduction in stress in response to a NE.

### Cortisol

A NE resulted in a 21.3%/h drop in cortisol beyond that of the hormone’s 11.7% diurnal drop. The efficiency of a nature pill per time expended was greatest between 20 and 30 min, after which benefits continued to accrue, but at a reduced rate.

#### Diurnal Response of Cortisol in Sample

**FIGURE 1:**
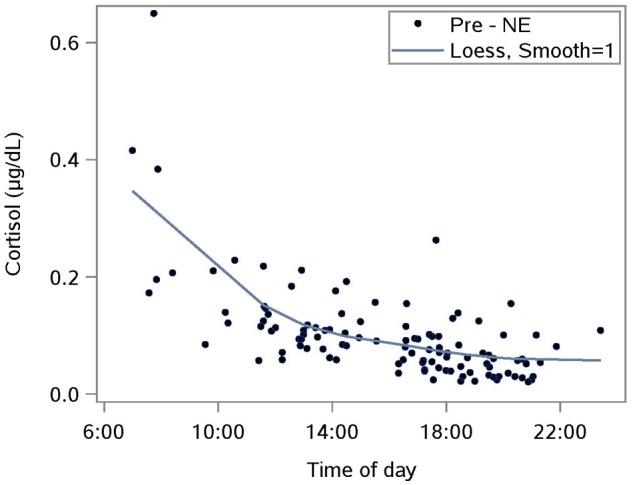
Characterizing the untransformed diurnal response of salivary cortisol using saliva samples taken prior to a nature experience (pre- NE); *n* = 110 NE measurements from 36 subjects; the smoothing parameter controls the flexibility of the Loess line.

The diurnal response was established with saliva samples collected just pre-NE. The Loess smoothing function was applied to untransformed data (Figure 1) for better visualization. The smoothed line shows a substantial drop in cortisol between waking and approximately 10 a.m., followed by a gentler decrease for the rest of the day. This pattern is consistent with published reports including one where subjects provided hourly saliva samples over the course of 1 day of normal activities (Nater et al., 2007).

**FIGURE 2:**
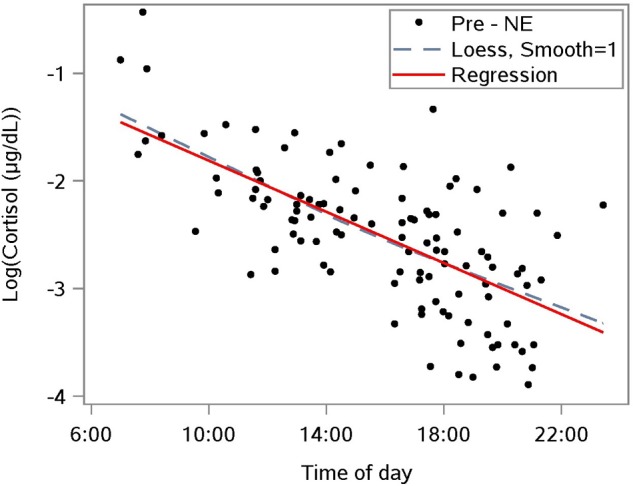
Diurnal response of the natural log-transformed cortisol from saliva samples taken prior to a nature experience (pre-NE); *n* = 110 NE measurements from *n* = 36 subjects. A linear mixed model accounts for repeated measures per participant and estimates the relationship as log cortisol = –0.641 – 0.117 ^∗^(time of day in hours); *p* < 0.0001 for slope; the predictor “time of day” explained 41.6% of level-1 (fixed effects) variance and 51.5% of level-2 (subject-level) variance.

To interpret the rate of change in cortisol over time, cortisol values were linearized by a natural log transformation (Figure 2). This transformation reduces the right-sided skew in the distribution of cortisol. A linear mixed model regression analysis of the transformed data estimated the diurnal drop in the sample population cortisol to be 11% per hour (*n* = 110) between morning and nightfall. This result provides a baseline for the diurnal drop, and enables us to distinguish the diurnal component of a change in cortisol level from a NE effect.

#### Separating Diurnal Response From Estimates of Nature-Based Stress Relief

The addition of a NE duration variable (length of a NE in minutes) to the mixed model of log cortisol on time of day (diurnal effect) shows that a NE produced a cortisol drop nearly two times greater than the average diurnal drop expected during the period of the NE (Table 1). After accounting for the 11.7% per hour diurnal drop in cortisol in this model, NEs accounted for an additional 21.3% per hour drop.

**Table 1:** Estimating the change in cortisol by duration of the nature experience (NE duration in hours), after accounting for the natural diurnal effect (time of day).

Effect	df^A^	Beta	Standard error	*p*-value	% Cortisol decrease per hour^B^
Intercept		–0.542	0.1952	0.0064	
Time of day (diurnal effect)	109	–0.124	0.01179	<0.0001	11.7%
Length of NE (NE duration)	128	–0.239	0.05815	<0.0001	21.3%

*^*A*^Denominator degrees of freedom ^*B*^Calculated as e ^*beta estimate*^ – 1. The diurnal effect is expressed as percent change per hour of log cortisol. Linear mixed models were used to account for multiple NEs per person (36 subjects, *n* = 220 saliva samples from 110 NEs). This model explains 26.9% of level-1 (residual – repeated measure) variance, 47.9% of level-2 (subject-level) variance, and 53.7% of level-3 (timepoint-level) variance.*

**FIGURE 3:**
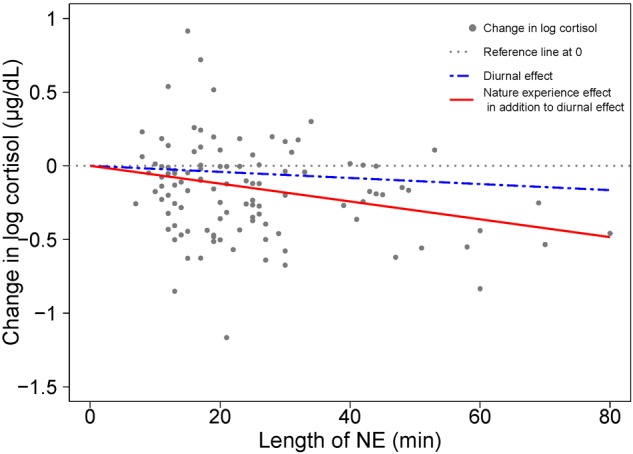
Visualizing the contributions to cortisol change from diurnal effects and nature experience effects based on model results in Table 1. A scatterplot shows the change in log cortisol levels (post-NE – pre-NE log cortisol) relative to duration (length of an NE in minutes); *n* = 110 NEs. The position of the horizontal dotted line indicates an absence of both diurnal and NE duration effects on cortisol. The blue dashed line shows the estimated diurnal decrease in cortisol (11.7% per hour) while the red solid line shows the estimated combined diurnal and NE duration effects on cortisol level. The difference between the blue dashed line for diurnal effect and the red solid line is the additional effect of the nature pill. The data points (gray dots) represent the observed change in natural log cortisol levels of study participants.

The role of duration on the magnitude of the cortisol response is visualized in Figure 3 as the degree of divergence between the two slopes – one for diurnal change and the other combining NE and diurnal effects over time. In the absence of a NE effect on cortisol, the coefficient for NE duration would be approximately zero, and the two regression lines would coincide. The divergence between the two regression lines indicates a NE effect on stress relief, as manifested by a cortisol drop.

#### Efficiency of a Nature Experience in Reducing Stress in Terms of Time Expended

To move closer to the goal of defining a reliable prescription, we used a step function model to locate the duration threshold for stress relief and the duration of greatest efficiency for stress reduction. A linear mixed model regression with change in log cortisol per hour = time of day (of saliva sampling) + NE duration interval (categorical) + NE duration (minutes) was fitted to the sample. Time of day for pre-NE samples captures the impact of a natural diurnal drop over the course of a NE. The NE duration interval variable, a categorical variable for the step function, was created with quartile partitioning. Because the NE start and stop times were reported in full minutes, the quartile partitioning is not at exactly 25%. The intervals shown in Table 2 gave the most equitable distribution of samples while accommodating the left-skewed distribution of the NE duration variable.

**Table 2:** Efficiency of a nature experience in reducing stress in terms of time expended.

Effect		*n*	Beta	Standard error	*p*-value	% Cortisol drop/hour^A^
Intercept		110	–0.52	0.197	0.009	
Time of day (diurnal effect)		110	–0.125	0.0119	<0.0001	11.7%

**Duration interval: length of NE (min) per quartile^B^**	**NE frequency for each minute in the interval**	***n*/% of total sample**				**% Cortisol drop beyond diurnal effect^A^**

Q1: 7–14 min	1,2,1,2,5,7,7,3	28/25.5%	–0.0864	0.0561	0.13	8.3%
Q2: 15–20 min	5,2,8,1,6,5	27/24.5%	–0.0375	0.0572	0.51	3.7%
Q3: 21–30 min	4,1,3,2,6,4,3,1,1,5	30/27.3%	–0.2048	0.0545	0.0003	18.5%
Q4:>30 min	1,1,2,1,1,1,1,2,1,2,1, 1,1,1,1,1,1,2,1,1,1	25/22.7%	–0.1214	0.0600	0.045	11.4%

*^*A*^Calculated as e^*beta estimate*^ – 1. ^*B*^Reported in minutes, calculated as proportion of an hour. Mixed models of log cortisol levels as predicted by diurnal effects (time of day) using a linear function and by duration of a nature experience using a step function. The step function estimates are calculated for each quartile interval (Q) separately and are not cumulative. This model explains 26.7% of level-1 residual [repeated measure] variance, and 45.9% of level-2 [subject-level] variance, and 53.4% of level-3 [timepoint-level] variance.*

A step function model of NE duration (Table 2) shows a significant reduction in the stress hormone cortisol after a NE greater than 20 min. Stress relief was most efficiently gained when a nature pill lasted between 21 and 30 min when cortisol dropped at a rate of 18.5% per hour beyond diurnal effects. Thereafter, benefits continue to accrue, but at a reduced rate of 11.4% per hour.

**FIGURE 4:**
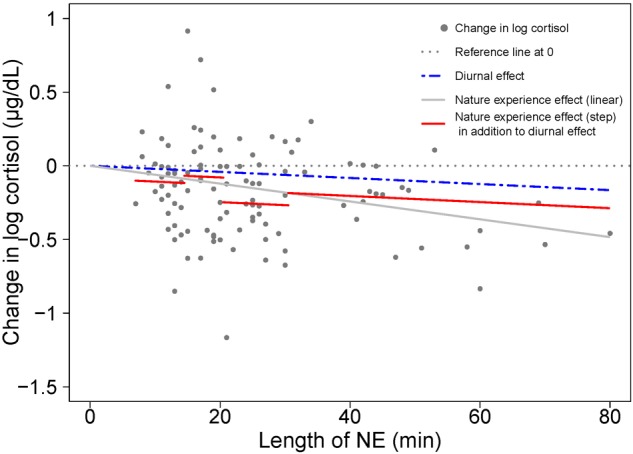
A visual comparison of cortisol response to NE duration with linear (gray solid line) and step function regressions (solid red line segments) based on the results shown in Tables 1 and 2. As in Figure 2, the blue dashed line represents the diurnal effect of change in log cortisol. The difference between the blue dashed line and the red solid line segments represents the nature experience effect in addition to the diurnal effect.

A visual comparison of linear and step function regressions for the cortisol response to NE duration provides insight on the nature of the variation in the linear model (Figure 4). Investigation using a step function offers a way to uncover the relative restoration efficiency of different nature pill durations and provides a guidance on optimal duration of a nature pill in terms of cortisol drop/stress relief efficiency.

### Amylase

A NE resulted in a 28.1%/h drop in amylase after adjusting for its diurnal rise of 3.5%/h, but only for participants that were least active sitting or sitting with some walking. Activity type did not influence cortisol response.

#### Establishing the Diurnal Response of Amylase

**FIGURE 5:**
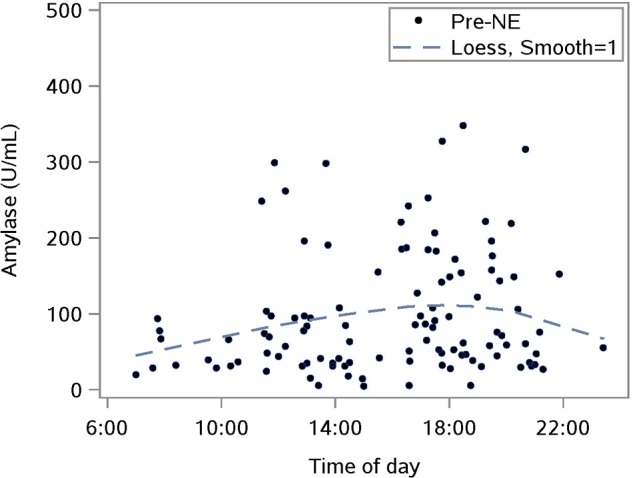
Characterizing the untransformed diurnal response of salivary amylase in our sample using pre-nature experience (NE) amylase levels for all NEs; *n* = 110 NEs from 36 subjects; the smoothing parameter controls the flexibility of the Loess line.

Salivary amylase levels measured pre-NE showed the expected diurnal form of untransformed data: a rise from morning to some point in the evening after which, amylase falls until morning (Figure 5).

After log transformation to normalize pre-NE amylase data (due to its right-sided skew), a linear mixed model for diurnal effect was fitted to the data (*n* = 110): log amylase = 3.8 + 0.027^∗^(time of day in hours). The 95% CI for the slope was (-0.0036, 0.05763), indicating that the slope was not significantly different from zero (*p*-value = 0.084). This was unexpected given the outcomes in other field studies showing a rising diurnal amylase response (e.g., Nater et al., 2013b). Our study differed from others by its inclusion of saliva samples from NEs completed after nightfall. Although the impact of day length on amylase production is unstudied in humans, its production changes when under different photoperiod treatments in rats (Bellavia et al., 1990). In our study, all NEs began sometime after dawn but seven of the 110 NEs ended after dark – in violation of the ground rules given to participants. Consequently, we tested a set of hypotheses to identify the time of day when the direction of amylase production shifted. This investigation became the basis for deciding which saliva samples would not be included.

#### Estimating the Time of Directional Shift in Diurnal Amylase Response

The investigation used two approaches to identify a reliable cutoff point for NE data inclusion based on the timing of the shift in the direction of diurnal amylase production. First, we compared the slopes from a set of linear mixed models using NE datasets that had cutoff times set one hour apart from 6 p.m. through midnight. This approach gave the opportunity to compare our results with those of several other studies that ended between 4 and 8 p.m. The mixed model with the largest slope (beta) came with NEs completed by 9 p.m. (Table 3). Nine in the evening was close to the average sunset time of all NEs – 9:04 p.m., during the experimental period that ran from June 17 (sunset at 9:14 p.m.) to August 21 (sunset at 8:47 p.m.).

**Table 3:** Mixed model linear regressions of pre-NE log amylase on time of day using eight subsets of the data, each differing by the latest time of the post-NE saliva sample.

Pre-NE samples from NEs ending as late as:	Beta	*p*-value	*n*	Subject number	Den df
6:00 p.m.	0.04640	0.1374	67	33	54.2
7:00 p.m.	0.04339	0.0681	79	34	61.2
8:00 p.m.	0.03149	0.0871	91	34	68.5
Sunset – time varies	0.03584	0.0230	103	35	78.7
9:00 p.m.	0.03253	0.0393	100	35	75.0
10:00 p.m.	0.02831	0.0622	108	36	84.1
11:00 p.m.	0.03064	0.0529	109	36	84.1
12:00 a.m.	0.02699	0.0835	110	36	88.9

*Each hourly interval includes all NEs that started after 7 a.m. and ended by the time shown. By contrast, the sunset model includes NEs that ended by the time of sunset on the date of the NE. Regression slopes and *p*-values are reported. Denominator (Den) degrees of freedom (df) reflect the amount of information for regression error in the correlated repeated measures data.*

Next, we considered the role of sunset time, specific for the date of each NE. Data on time of sunset (civil twilight) in Ann Arbor, MI, United States, in 2014 came from http://www.sunrisesunset.com/usa/Michigan.asp. The best linear fit for the amylase diurnal response came from the data set delimited by a sunset criterion: post-NE saliva samples were taken before the time of sunset on the NE date (Table 3). Consequently, the remaining analyses of amylase response include only those 103 NEs that ended before sunset. It is of note that a comparable test with cortisol data showed that inclusion of the NE’s happening post sunset had no influence on the diurnal trajectory of falling cortisol based on model fit. This was expected as there is no shift in the direction of diurnal production of cortisol until a rapid escalation upon morning rising (Hadlow et al., 2014).

**FIGURE 6:**
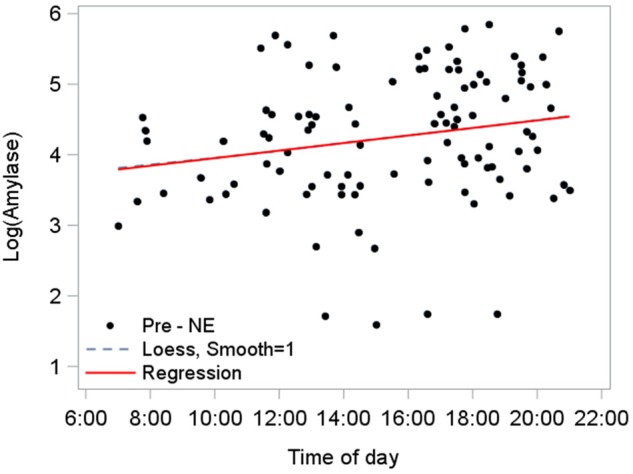
Diurnal response of amylase for NEs completed by sunset (*n* = 103). A mixed model accounts for repeated measures per participant and estimated the relationship as log amylase = 3.7 + 0.036^∗^(time of day in hours), 95% CI for slope (0.0051, 0.067), *p* = 0.023. The predictor “time of day” explained 4.3% of level-1 (fixed effects) variance and 4.8% of level-2 (subject-level) variance. The position and flex of the Loess line (dashed) nearly mimics the path of the model’s regression line, corroborating the model fit.

We established the diurnal baseline response of amylase using pre-NE saliva samples from NEs that ended before sunset (*n* = 103; Figure 6). The diurnal rise in amylase over the day until sunset was estimated at 3.7%/h, based on the slope (0.036) from the linear mixed model fitted to pre-NE log amylase: (e ^0.036^-1).

#### Effect of Different Types of Physical Activity on Amylase

No significant effect of NE duration on amylase was detected in a linear mixed model that controlled for diurnal effect (*p* = 0.21, beta = -0.116, *n* = 103 NEs). The discrepancy between this result and that of the cortisol biomarker led us to reconsider a key assumption of the amylase model: that activity type did not differentially influence amylase response. Note that most research on the amylase-physiological stress relationship focuses on athlete training and implies that amylase is elevated only with more intense activity types (Koibuchi and Suzuki, 2014; Peinado et al., 2014). The following paragraphs reveal how we determined that (a) sitting and sitting+walking during a NE produced very similar outcomes in terms of amylase production despite sample size differences; (b) amylase production in the non-aerobic walkers was extremely different than amylase production in the two sitting groups; and (c) the amylase response when sitting or sitting+walking was very similar to that of the stress hormone cortisol (which is not sensitive to activity type).

To test our assumption, we needed to determine whether participant and activity type were confounded in this repeated measure design. We found that 36 participants did not choose the same activity type for each NE: 21 participants engaged in 31 “sitting” NEs, 12 participants engaged in 17 sitting+walking NEs, and 23 participants engaged in 55 “walking” NEs. The average diversity of activity types per individual was 1.64 out of 3 (SE = 1.05); 44% stuck with one activity type, 47% used two types, and 8% used all types.

Next, we looked for differences in amylase response as a function of activity type. A linear mixed model with a three-category covariate for activity type (sitting; sitting+walking; walking only) was fitted to the log transformed amylase data (Table 4, Model 1). Activity type was associated with a notable difference in amylase response (with diurnal changes accounted for): a walking NE produced a 4% drop per hour versus a sitting NE (34% drop per hour) or a sitting+walking NE (30% drop per hour).

**Table 4:** The effect of physical activity type on amylase.

Activity type (# participants, #NEs)		Time of day (h)/diurnal effect		Length of NE (h)/duration	
	*n*	Beta	*p*	Beta	*p*
**Model 1. Activity levels**					
Sitting (21, NE = 31)	62	0.034	0.013	–0.34	0.08
Sitting + walking (11, NE = 17)	34			–0.30	0.30
Walkers (23, NE = 55)	110			–0.04	0.70
**Model 2. Activity levels grouped**					
Sitting and sitting + walking (NE = 50)	96	0.034	0.013	–0.33	0.047
Walkers (NE = 55)	110			–0.04	0.702

*Mixed model linear regressions of log amylase on time of day (diurnal effect), duration of the nature experience, and activity type during the NE. Model 1 includes three activity levels; Model 2 collapses “sitting” and “sitting + walking” to distinguish the two lower activity groups from the highest activity group “walkers” (*n* = 206 amylase measures from 103 NEs).*

**FIGURE 7:**
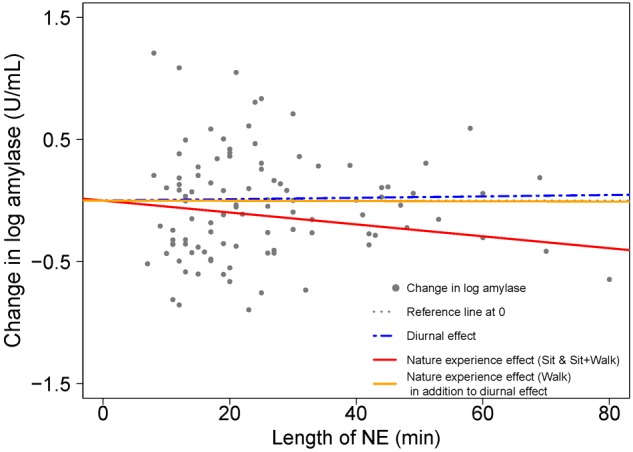
Visualizing the contributions to amylase change from start to end of the NE due to diurnal effects and nature experience effects for two activity types; based on model results in Table 4. A scatterplot shows the change in natural log amylase levels (post-NE – pre-NE) relative to duration (length of an NE in minutes); *n* = 103 NEs. The position of the horizontal dotted line (black) indicates an absence of diurnal and NE duration effects on amylase. The blue dashed line estimates the diurnal increase in amylase (+3.5%/h) while the solid lines (red for NEs while sitting and yellow for NEs while walking) estimate the combined effects of the circadian cycle and duration of an NE on amylase level. The difference between the blue dashed line for diurnal effect and the red solid line is the additional effect of the nature pill. The data points (gray dots) represent the observed change in natural log amylase levels of study participants.

Next, we grouped data from the two lowest exertion classes (sitting and sitting+walking) based on the similarity of the amylase responses (i.e., regression slopes). The linear mixed model with a two-category covariate for activity type (Table 4, Model 2) showed that lower exertion nature pills had an amylase drop of 27.9% per hour (with diurnal changes accounted for). Higher exertion nature pills had amylase changes that were indistinguishable from the baseline diurnal condition. Figure 7 visualizes these relationships.

Unlike cortisol, amylase data could not be used to estimate what duration period was most efficient for stress reduction because separation by activity type led to inadequate sample size. It is important to point out that the regression of cortisol on NE duration produced the same slope for low and high exertion categories (Table 5). Based on a comparison of low exertion conditions such as sitting (Table 6), we conclude that the NE-based decline in cortisol and amylase are comparable when a nature pill is taken, but that cortisol is a more robust biomarker for field studies.

**Table 5:** Effect of physical activity type on cortisol.

Activity type (# participants, #NEs)		Time of day (h)/diurnal effect		Length of NE (h)/duration	
	*n*	Beta (SE)	*p*	Beta (SE)	*p*
Sitting (21, 33) and Sitting+walking (11, 17)	100	–0.124 (0.012)	<0.0001	–0.240 (0.010)	0.023
Walkers (23,60)	120			–0.239 (0.068)	0.0006

*Mixed model linear regression of log cortisol on time of day (diurnal effect), duration of the nature experience, and two activity groups (*n* = 220 from 110 NEs).*

**Table 6:** Rate of change in two stress biomarkers per hour due to underlying circadian rhythm (diurnal effect) and activity types; based on model outcomes for cortisol in Table 5 (NE = 110) and for amylase in Table 4, Model 2 (NE = 103).

Effect	Model beta	% Cortisol change/hour	Model beta	% Amylase change/hour
Diurnal effect	–0.124	–11.7%	+0.034	+3.5%
NE duration while sitting or sitting + walking	–0.240	–21.3%	–0.33	–28.1%
NE duration while walking only	–0.239	–21.3%	–0.04	–3.9%

*For negative slopes, % drop is calculated as (1 – e ^*beta estimate*^); for positive slopes, % rise is calculated as (e ^*beta estimate*^ – 1).*

## Discussion

The field study presented here offers the first estimates of the impact of NE duration on stress level in the context of normal daily life. Spending time with nature produced a significant drop in the stress hormone cortisol, with the duration of the NE contributing to the amount of stress reduction. The research also breaks new ground in the following ways, primarily by addressing some of the complexities of measuring an effective nature dose. First, it directly investigates the duration aspect of a successful nature pill prescription in the context of real life. Second, the 8-week field experiment enabled repeated measures of each participant in different contexts, thereby allowing the circumstance of an individual’s daily life into prescription development of the nature pill. Third, an adaptive management approach allowed participants to self-manage the time, place, and duration of each nature pill to offset the inevitable challenges of scheduling a non-essential activity. Fourth, a novel approach to data evaluation offers a way to distinguish nature pill effects from diurnal effects without repeated invasive saliva sampling for baseline physiological status throughout the day of an NE. Finally, the results provide a validated starting point for healthcare practitioners prescribing a nature pill to those in their care.

### Cortisol

#### Cortisol Response: Recommendation for a Nature Pill Prescription in Terms of Duration

Two models with good fit investigate the trajectory of cortisol in terms of duration of the NE. The linear model of change in log cortisol on NE duration predicts a stress reduction of 21.3% per hour. Because people find it difficult to make time for self-care, a step function model asked the question: what is shortest duration needed to achieve benefit? We found that stress relief is significantly and most efficiently gained (18.5% cortisol drop/h) when the nature pill lasted between 20 and 30 min, and significant benefits continued to accrue thereafter at a somewhat reduced rate (11.4%/h). This is a useful and robust starting point for a nature pill script because it emerged from an adaptive management platform, embracing the typical variation in how people make or use their free time. Needless to say, the true functional form of the response will require further testing with a larger sample size, an expansion of the duration times (especially < 10 and 31–60 min), and sample populations with a broader age and gender representation.

#### Distinguishing Nature Pill Effects From Diurnal Changes in the Stress Markers

A population response to NEs is a useful basis for defining parameters of a nature pill prescription where parameters are largely controlled by the participant rather than the researcher. We were able to evaluate the impact of a NE *in situ*, using a population approach to provide a reliable estimate of the baseline diurnal pattern of each stress marker. Mixed model regression to handle repeated measures could distinguish the difference between an expected change in stress markers (diurnal effect) and realized change in stress markers at the end of a NE (NE-based effect).

Accurate assessment of diurnal drop of cortisol is key to accurate assessment of a nature pill effect. Further support that our diurnal cortisol drop of 11.7% per hour is robust comes from the results of three repeated measures studies with similar outcomes to ours, despite differences in experimental goals. In all cases, participants went about their daily life during the study. The key difference between our study and the studies described below is that our study estimated the diurnal response of cortisol with a single saliva sample (pre-NE sample) per participant on each sampling day. The other studies estimated diurnal cortisol with four to five saliva samples per participant on each sampling day.

Laudenslager et al. (2013) reported an 11.0% per hour drop in diurnal cortisol over a period of 10 hours beginning 30 min after waking (*p* < 0.0001; 95% CI: -13, -9%). The study was focused on testing a novel saliva collection device to support participant-controlled field sampling. The study had a similar sample to ours: 32 participants, 81% female, 18% were not Caucasian, and the age range was roughly the same as our study. The only restriction on participants’ behavior was to avoid eating, teeth brushing, and drinking liquids within 15 min before saliva sampling. The design used repeated measures: each participant collected saliva for 3 consecutive days at four time points in the day. A linear mixed model regression included log transformed cortisol data and collection time of day. There was some allowed flexibility in the collection time of saliva: at awakening (flexible), 30 min post awakening, just before lunch (flexible), and 10 h after waking. Compliance with collection time for the second and fourth sample of the day was not perfect, yet tolerance testing for 7.5 and 15 min offsets revealed no significant effect.

In a study of quality of life in relation to demographic and socioeconomic differences, Karlamangla et al. (2013) analyzed data from 1693 participants whose ages fell within the same range as our study; 57% female and 86% Caucasian. Participants provided saliva samples on 4 days over a week’s period that included both weekdays and weekend days. On each sampling day, saliva was taken: at awakening (flexible), around 30 min post awakening, just before lunch (flexible), and at bedtime (flexible; median bedtime was 10:30 p.m.). This produced significant variability in actual time of sampling and allowed examination of cortisol level across the entire day in the sample population “to get a general idea of the shape of the mean daytime cortisol trajectory” (pg. 4). They reported a cortisol diurnal drop of 8.1% (beta = -0.084) for a 10.5 h period that began 4.5 h after rising. This temporal division of data was enabled by a high sample size (>24K cortisol data points), and the use of the more flexible linear spline model. Their model was also indexed on “time since waking” and adjusted for a participant’s average length of waking day, length of sleep the previous night, waking time on day of measurement, and weekend versus workday status.

Nater et al. (2013b) report a mean diurnal cortisol drop of 12.2% (beta = -0.13) in an investigation of diurnal cortisol and amylase profiles over adult life span. The study included 185 participants (median age of 49, 51% female, 74% Caucasian) who provided a saliva sample five times per day on each of seven test days over a 10-day testing period. The estimate of mean diurnal cortisol drop was based on the difference in cortisol level at waking and the last sample of the day (9:00 p.m.), taking respective time of day into account. Sample times throughout the day were: at awakening, 30 min later, 9 a.m., 12 p.m., 3 p.m.,6 p.m., and 9 p.m. At these times, participants provided a saliva sample and answered a questionnaire.

In light of these comparisons, we conclude that our experimental design and analytical approach offers an efficient way to investigate the value of self-care behaviors in preventive health care that rely on adaptive management. Our approach is also an alternative to experimental designs that require more control over participant behavior, such as pharmacology dose–response or clinical trial testing. We recognize that our experimental approach is possible largely because the nature pill intervention is not dangerous if misused. The relative safety of a nature pill in any dose is one reason that health care professionals around the country feel comfortable prescribing nature pills to patients without the benefit of guiding data.

#### Comparison of Cortisol Results From Other Outdoor Studies of the Nature Effect

The results of our repeated measures study can be considered in light of field studies that use cortisol to assess the impact of single episodes of nature exposure on psychological stress. Japanese studies of Shinrinyoku – immersing oneself in nature by mindfully using all five senses (Tsunetsugu et al., 2010; Hansen et al., 2017) have provided recurrent support for the ability of nature to reduce stress. The protocols are well considered and meticulous and are akin to those of most other field tests of nature’s stress reducing potential.

Park et al. (2010) complied data from many studies done under exacting conditions that compared salivary cortisol levels of male college students (*n* = 280) who spent time in forest and nearby urban settings (the control) in 24 National Forests of Japan. Subjects were housed in controlled settings on the nights preceding testing days. In each National Forest and at the same time of day, 12 participants spent 15 min sitting while viewing either the forest (*n* = 6) or a nearby urban setting (*n* = 6). Participants were tested likewise on a consecutive day in the alternative setting (random crossover trial). Before and after a 15-min test interval, saliva was collected (comparable to our method). A pairwise means comparison of the two treatments (forest versus urban setting) showed that the average salivary cortisol level of participants was 13.4% lower after 15 min of forest viewing while sitting compared to urban viewing. For just over a third of the sample group (∼75), the 15-min sitting period was preceded by a 15-min walking period in the forest. Cortisol was 15.8% lower after 15 min of forest walking compared to participant’s response to walking in the urban setting. Diurnal effect was ignored as testing time was approximately the same for both treatments. Using the same experimental approach, Park et al. (2012) expanded the database to include 420 participants at 35 different forests throughout Japan. They reported that participants walked their assigned areas for 16 ± 5 min, then sat and viewed the area for 14 ± 2 min. They reported a 12.4% drop in salivary cortisol after the forest experience compared to the urban experience. Kobayashi et al. (2017) point out that although the time of saliva collection varied from 9 a.m. to 12 p.m., each participant was measured at approximately the same time on each experimental day for both environments.

Unlike the Shinrinyoku research, our study yields NE outcomes in terms of an hourly rate of stress reduction after diurnal effects are taken into account. Our linear model reveals the overall stress reduction of cortisol at 21.3% per hour. Interpolation from this model predicts a 10.6% cortisol drop after a 30 min NE. By contrast, the Shinrinyoku studies report a 12–15% cortisol drop after a 15-min period of forest sitting. We consider these outcomes quite similar considering that several modifiers are likely in play. Acclimation to field setting: Shinrinyoku participants spent time getting accustomed to the setting before the first cortisol sample was taken (walking from transport to the field site and spending time at the field site for several other physiological measures). Additionally, participants had a low stimuli common experience for at least 12 h preceding the field test. By contrast, our experiment had significantly lower level of control over participant behavior before and during the field tests because of the adaptive intervention strategy. Quality of the “nature” setting: Shinrinyoku took place in national park settings which brought sensory continuity to the experience of all participants and offered a consistently high opportunity for a sense of nature immersion. By contrast, there was variation in our participant’s choice of each NE setting, typically in urban green space near enough to be convenient. Finally, Shinrinyoku testing was done over 2 days with participants removed from their daily routine and led under the direction of researchers versus the adaptive management situation that requires a self-motivated decision to take a nature break when time permits within daily life. Overall, we think that the results of our adaptive intervention dose–response study are coherent with other studies given the differences outlined above. The source of these differences also makes clear the need to move forward with a focus on additional dose–response studies that use comparable methods to identify minimum dose recommendations for specific health outcomes.

### Amylase

#### Amylase Response: Corroboration With NE-Based Cortisol Response

It is of note that our amylase results for the impact of a NE on stress corroborate those of cortisol. When low exertion nature pills were evaluated, the degree of restoration indicated by amylase (28.1% per hour, *n* = 50 NE) is comparable to that of cortisol (21.3% per hour, *n* = 110 NEs). The slightly larger restoration value for amylase may be related to differences in time of production and release of these stress markers. In a study of response to acute stress, salivary alpha-amylase response was faster than cortisol (Takai et al., 2004).

As with cortisol, our analytical approach required a reliable estimate of the amylase diurnal baseline. Our study showed a 3.5% rise in amylase from 1 h after rising until sunset. We could find only two relevant studies reporting diurnal amylase response in terms of a slope. Both gave results comparable to ours. Nater et al. (2013a) report a mean diurnal amylase rise of 4.1% (beta = +0.04) in an investigation of diurnal cortisol and amylase profiles over adult life span. Details about this work are given above (section “Amylase”).

Out et al. (2013) reported a 2.15% increase in salivary amylase from waking to evening in a repeated measure study (*n* = 122 participants) involving five saliva samples per day for each of 3 days in five sampling periods between August and February. These data also indicated that variation in diurnal response within an individual varied by less than 1%. From this, Out et al. (2013) and others cited in their paper suggest that diurnal amylase profile is relatively stable compared to the response to momentary stress and ongoing moderate stress of both physical and psychological origin.

#### The Utility of Using Salivary Amylase for Studies of Nature-Based Stress Relief

The inclusion of salivary amylase in this NE study adds to the growing body of information about the value of using this stress marker in restoration studies. Using amylase to evaluate calming interventions such as communing with nature uncovered two confounding effects: the sensitivity of amylase to physical exertion and time of sunset.

#### Amylase Sensitivity to Time of Sunset

Our data define a diurnal rise in amylase of 3.5% per hour over daylight hours, based on an 8-week study period in summer. The sampling period was confined to 1 h after rising to time of sunset, eliminating those samples from participants who bypassed the request to take the nature pill “before dark.” The data analysis ultimately included only those NEs that ended before sunset time on the NE date. These criteria led to the best model fit for linearity in the log-transformed data (Table 3). The decision to use sunset time as the cut point for data inclusion gains support from other research studies that did not detect a problematic point of downturn in diurnal amylase during the evening hours. For example, the latest time of day for sampling include 8 p.m. (Nater et al., 2007), 9 p.m. (Out et al., 2013), and (unspecified) bedtime (Karlamangla et al., 2013). Nonetheless, all of these results underscore the need to control for time of day when doing research where amylase is the stress marker.

The role of sunset time through the year on diurnal cycle of alpha-amylase needs formal investigation. At present, we know of only one other study, with rats, indicating an effect of sunset time on alpha-amylase. Bellavia et al. (1990) reported that the diurnal pattern of production disappeared when photoperiod changed to constant light or constant dark for 15 days. Based on our results, we hypothesize that time of year and latitude, both of which contribute to day length and sunset time, will adjust the temporal form of diurnal amylase production. It is particularly important to learn more about the role of day length variation in studies about nature restoration because evening often affords working people greater flexibility for self-care. New research on the after dark trajectory of amylase diurnal production is also of great interest because an after-dark nature pill affords the opportunity to investigate non-visual aspects of nature restoration, a critical but relatively unstudied aspect of our relationship with the environment (Franco et al., 2017).

#### Amylase Sensitivity to Physical Exertion

We were somewhat surprised that casual walking produced such a noticeable effect on salivary amylase production. In terms of this stress marker, the drop in amylase after a walking nature pill was small (4% per hour) and indistinguishable from the diurnal baseline. By contrast, under low exertion – sitting or sitting with some walking, the nature pill produced a 28% per hour drop in amylase after accounting for the diurnal baseline. In a literature review that focused on high intensity exercise, Koibuchi and Suzuki (2014) concluded that exercise upregulates salivary amylase. The few studies that involved low intensity exercise did not show amylase elevation: a 30-min light gymnastics program for the elderly and relaxed 20-min walks in either forest or urban settings by university students. In our experiments, the assumption that non-aerobic walking would not constitute a physical stressor was wrong.

In another experiment using self-report data on psychological response rather than physiological response, the impact of exercise intensity also showed up. Barton and Pretty (2010) evaluated the effect of green exercise (activity in the presence of nature) on subjective ratings of mood and self-esteem from over 1200 participants, each involved in one of 10 experiments carried out over a 6-year period in the United Kingdom. Estimates of dose–response relationships in terms of green exercise duration and intensity showed significant benefits to mental well-being after even short engagements with green exercise – as little as 5 min, with positive effects diminishing over duration periods up to a half day, then rising with durations up to a full day. Improvements to self-esteem and mood during their first duration period (less than an hour – a duration that is coincidental with that in our experiment) were greater for the lower intensity green exercise (e.g., walking) compared to more intense exercise (e.g., cycling).

### Limitations and Potential

To develop a robust basis for prescribing a nature pill, more research is needed on the role of NE duration, frequency, and the perceived quality of the nature experienced (beyond the natural versus urban environment dichotomy) in the delivery of positive effects. Key among the challenges is the need for a large and diverse sample size because the diurnal form of cortisol and amylase production changes with age and stress level (Strahler et al., 2017), socioeconomic factors (Karlamangla et al., 2013), and lifestyle factors such as sleeping patterns (Van Lenten and Doane, 2016). A truly functional form of the nature pill prescription will emerge from testing a more diverse participant pool (gender, age, and lifestyle) from a diversity of settings (habitat types, both familiar and novel), and across seasons. The experimental approach described here can efficiently support the large sample size needed to accommodate these factors.

Researchers have discussed the difficulty with adherence in prescribed behavior testing (Olem et al., 2009; McCahon et al., 2015). In our experiment, we balanced stringency with adaptive adherence opportunities in order to get a realistic assessment of the value of NEs under normal circumstances. For example, the requirements to make time for repeated NEs with limitations on ingesting, social exchange, and aerobic exercise, etc., were balanced by the freedom choose when, where, and duration (beyond 10 min) of a NE. That said, the use of self-selected participants who were more likely to adhere to the parameters of the experiment could have biased the results if the benefits of nature are more readily gained by those who are willing to spend or enjoy spending their free time in this way. Future experiments would benefit from a participant group that showed the range of interest in NEs but were equally rewarded (e.g., money) for adherence regardless of natural inclination.

Our study had 36 participants, a sample size that is twice that of the median sample size in 18 studies on the same topic (see review by Kondo et al., 2018). However, our sample size for the number of nature pills with a saliva collection was not sufficient to fully investigate duration times at either end of the spectrum (i.e., under 10 min and over 30 min). Additional research is needed on this aspect of nature pill duration.

The usefulness of amylase in this study was reduced by confounding effects of physical exertion and time of sunset. These effects can be handled through experimental design and *post hoc* data cleaning. But why use amylase when cortisol is without these limitations? There is opportunity for efficient and cost-effective self-monitoring of amylase with technologies that are already in the marketplace. For example, amylase can be readily measured in the field using a phone app and an add-on sampling device that attaches to the phone (Zhang et al., 2015). Such devices are valuable for athletic training because exertion stress data (e.g., aerobic threshold) can be used to formulate an effective training plan (Akizuki et al., 2014). To be useful for tracking mental stress, however, it would appear that low physical exertion is required. To demonstrate the ability of their smart-phone-based potentiometric biosensor to test psychological status, Zhang et al. (2015) evaluated salivary amylase in sitting participants before and after exposure to images from the affective picture system (IAPS), known to induce positive and negative emotions. The outcome was in good agreement with a published report of the same test using traditional collection and sample analysis methods for amylase level.

Our experimental approach for assessing the restorative power of a NE offers an efficiency and clarity that can be used to better address questions about duration, frequency, attenuation, and the efficacious quality of the nature encountered, particularly in an urban setting (Bratman et al., 2012; Hunter and Askarinejad, 2015; Cox et al., 2017a; Frumkin et al., 2017). Answers to these questions will also support better-informed economic models and policy decisions aimed at containing personal, societal, and health care costs (Kardan et al., 2015; Wolf et al., 2015; Shanahan et al., 2016) and, ultimately, support the cultural uptake of more time outside/less time on-screen^4^.

## Conclusion

The methods for this adaptive management study of nature-based restoration break new ground in addressing some of the complexities of measuring an effective nature dose in the context of normal daily life. Our approach was empirically field tested in the service of measuring the relationship between the duration time of a NE and stress level using physiological biomarkers. The stress markers revealed that taking a nature pill reduces stress by 21%/h (salivary cortisol) and 28%/h (salivary amylase). When the duration of the NE is between 20 and 30 min, the gain in benefit is most efficient.

This work is novel in several ways.

(1) Results came from an experimental approach that can efficiently distinguish the contribution of a nature-based stress reduction from the concomitant diurnal change of a stress marker.
(2) The experimental design bypasses the need for participants to take multiple saliva samples throughout each testing day to establish a personal diurnal curve. Instead, one saliva collection (pre-NE) per sample date established a diurnal form of the stress markers that was comparable to the diurnal trajectory in other outdoor studies.
(3) Unlike previous studies, this one included repeated-measures testing of the same individuals over 2 months, allowing us to capture the realism of changing psychological and physiological states and reactions to changing environmental context for each participant.
(4) The experimental format is unique for nature restoration research in its use of an adaptive management design. Participants had significant control in how they “medicated” themselves in terms of when, where, and length of a NE. This flexibility is essential for establishing and maintaining self-care behaviors in the face of responsibilities to others, lifestyle, and personal preference.
(5) The data analysis demonstrates how to quantify parameters of a nature prescription using a population approach for evaluation of nature exposure along a duration continuum set by participants.

The outcomes of our experiment are coherent with those of studies of stress biomarkers involving much greater control and much higher sample sizes. Moreover, the empirical results on stress reduction relative to the duration of a NE offer a validated starting point for healthcare practitioners prescribing a nature pill to those in their care. We think that our methodological approach for parameterizing a prescription (duration, frequency, and nature quality) for the nature pill is a tool that can be used by a field of study poised for new insights on the contributions of age, gender, seasonality, physical context, and cultural context to the effectiveness of nature exposure on well-being.

## Ethics Statement

This study was carried out in accordance with the recommendations of the Institutional Review Board of the University of Michigan, IRB-Health Sciences and Behavioral Sciences (HSBS) committee, with written informed consent from all subjects. All subjects gave written informed consent in accordance with the Declaration of Helsinki. The protocol was approved by the Institutional Review Board of the University of Michigan, Ann Arbor, MI, United States (IRB # HUM00089147). Regarding exemption status, the IRB committee has also determined that the study, as currently described, is now exempt from ongoing IRB review, per the following federal exemption category: research in which study activity is limited to analysis of identifiable data. For purposes of this research study, all research subject interactions and interventions have been completed and the data continue to contain subject identifiers or links. The research is not federally funded, regulated by the FDA, or conducted under a Certificate of Confidentiality.

## Author Contributions

MH made substantial contributions to the conception of the work, the experimental design, the acquisition of data, the analysis and interpretation of data, and drafting the manuscript and agreed to be accountable for all aspects of the work in ensuring that questions related to the accuracy or integrity of any part of the work are appropriately investigated and resolved. BG made substantial contributions to the analysis and interpretation of data and revising the manuscript critically for important intellectual content. SC made substantial contributions to the analysis of data and revising the manuscript critically for important intellectual content.

## Conflict of Interest Statement

The authors declare that the research was conducted in the absence of any commercial or financial relationships that could be construed as a potential conflict of interest.

## Footnotes

1 moodwalks.ca

2 naturesacred.org

3 http://www.sunrisesunset.com/usa/Michigan.asp

4 https://www.apha.org/policies-and-advocacy/public-health-policy-statements/policy-database/2014/07/08/09/18/improving-health-and-wellness-through-access-to-nature
